# Retraction Statement: Atypical parkinsonism and self‐mutilation: A new lens on the old concept

**DOI:** 10.1002/ccr3.7089

**Published:** 2023-03-26

## Abstract

Salari, M, Etemadifar, M, Ghanbari, K. Atypical parkinsonism and self‐mutilation: A new lens on the old concept. Clin Case Rep. 2021; 9:e04432. (https://doi.org/10.1002/ccr3.4432).

The above article, published online on 09 July 2021 in Wiley Online Library (wileyonlinelibrary.com), has been retracted by agreement between the authors, the journal Editor‐in‐Chief Charles Young, and John Wiley & Sons Ltd. The retraction has been agreed upon due to an error at the publisher, which caused a duplicate of the article to be published. The correct version of the article is Salari, M, Etemadifar, M, Ghanbari, K. Atypical parkinsonism and self‐mutilation: A new lens on the old concept. Clin Case Rep. 2021; 11:e04958. (https://doi.org/10.1002/ccr3.4958).
